# Bibliographical Notices

**Published:** 1853-10

**Authors:** 


### BIBLIOGRAPHICAL NOTICES.

---

*A Practical Treatise on the Diseases of Children*, by J. Forsyth Meigs, M. D., Lecturer on the Practice of Medicine in the Philadelphia Medical Association, etc., etc. *Second Edition, Revised and Enlarged.* Philadelphia, Lindsay & Blakiston, 1853. pp. 711.

This work is a republication, with considerable additions and modifications, of one of the series of "The Medical Practitioner's and Student's Library." The original edition having been exhausted for some time, a second has now been brought before the profession as a separate and distinct treatise. The additions consist of an Introductory Essay on the Clinical Examination of Children, and an article on Atelectasis Pulmonum, or imperfect expansion of the lungs. The articles on Croup, Bronchitis, Pneumonia and Scarlet Fever, have been entirely re-arranged and in many parts re-written; that on Measles has been much added to; and the subject of Tracheotomy in Croup has been discussed at considerable length.

The essay on the clinical examination of children is a very proper, we might almost say, a necessary introduction to a work of this kind. The diagnosis of infantile affections is replete with difficulties to the young practitioner, and these can never be overcome unless he knows what to observe and how to conduct his examination. The whole article will repay a careful perusal. We would particularly commend to the reader's attention, the passages under the head of "The Cry," and "The state of the cutaneous surface," as containing much valuable information. It is evident that Dr. Meigs has paid great attention to the subject of Semeiology. His Essay is not a mere digest of the opinions of authoritative writers upon the subject, but is replete throughout with remarks which show accurate and careful observation.

No physician of the present day should be ignorant of the important part, Atelectasis plays in the diseases of children. Its frequent occurrence in early childhood from various causes, and its connexion with Catarrh and Bronchitis, are admitted by all who have studied the subject. Dr. Meigs makes two divisions of the affection. 1. Congenital Atelectasis, in which larger or smaller portions of the lung have never been penetrated by the air: a continuation, in fact, of the fœtal condition; and 2d, Collapse of the lung or post-natal Atelectasis. As a sample of his mode of treating the subject, we extract the following:

"When, as indeed, most usually happens, collapse occurs in the course of bronchitis, it is associated of course with the symptoms of that disease. The bronchitic symptoms have lasted in their usual form for several days, having been marked by sonorous, sibilant, and subcrepitant râles, when suddenly, or in the space of a few hours, the breathing becomes much worse, the pulse rises in frequency but becomes small and feeble, and certain changes take place in the physical signs which are very important. The subcrepitant râle continues to be heard, but it is associated now with prolonged expiration, and a little later with bronchial respiration, which, however, is of a different kind from the bronchial respiration of pneumonia, being distant and smothered, instead of near and metallic, as in that disease. The percussion becomes at the same time, dull and obscure, but never, scarcely, to the same extent as in pneumonia. The general symptoms are those of exhaustion, rather than of high reaction. The surface is pale or slightly bluish, the skin is either natural, slightly warmer than usual, or coolish, the strength is very much reduced, and the child appears more seriously ill, and particularly more oppressed often than the amount of bronchitis present would seem to explain."

The causes, symptoms and anatomical appearances of both forms of the affection are described with great fullness and precision. In the treatment of collapse, he recommends warmth, nutritious diet, the use of mild stimulants and tonics, and the external application of revellents. When it occurs in the course or towards the termination of severe bronchitis, in addition to the above, he advises the application of dry cups or mustard poultices to the chest, and the use of emetics two or three times daily, unless they are found too exhausting. Electricity and insufflation of the lungs are not mentioned. In regard to the latter, MM. Rilliet and Barthez state that it is difficult to imagine that it can be hurtful in Atelectasis. They state their belief also, founded on Joerg's experiments, that carefully used in the first few days of the disease and following the inspiratory movements, it would be able to restore the lung to its normal condition. In the paroxysmal form of the disease, where other means had been used unsuccessfully, we should think it well worthy of a trial.

Dr. Meigs fully adopts the views of MM. Legendre and Bailly, regarding the nature of what was formerly termed lobular pneumonia. Their researches, with those of Dr. Gairdner, and his own investigations of the subject, have convinced him that in the great majority of cases, the affection in question was bronchitis complicated with collapse of the lung and not inflammation of the pulmonary parenchyma.

In relation to the views held by writers previously to the discovery of the true pathology of this disease, he says:

"The peculiar character of the lesions met with in many of the supposed cases of pneumonia, had often drawn attention and been commented upon, before their real nature came to be understood. The points of difference between these alterations and those of true pneumonia, were particularly noticed by MM. Denis, De La Berge, Rufz, and MM. Rilliet and Barthez, and Dr. West. In fact, M. Rufz, and MM. Rilliet and Barthez, both approached very near the truth in regard to these lesions, each comparing them, but the former at an earlier period than the latter, to the condition of the lung of a fœtus that has never breathed. The latter writers, in their article on pneumonia, have described a condition of the lung which differed so much from ordinary pneumonia, as to create a great difficulty in their minds as to its true nature, and to it they applied the term carnification. They were on the very verge of detecting its real character; they did in fact suggest its real character, but were so possessed with the idea that it must be the result of some inflammatory action, as to neglect to pursue their own suggestion, but endeavored to explain the condition on the ground that it was 'one mode of termination of pneumonia or else chronic pneumonia.' The following passage, quoted from their work (T. 1, p. 74), will show how closely they approached the truth: 'The first idea that enters the mind on examining this tissue (carnification), is, that it resembles the lung of a fœtus that has not breathed; we should feel inclined to say that the pulmonary vesicles had not yet been dilated under the influence of the thoracic expansion, and had not, therefore admitted air into their interior, or rather, it would seem as though they had been obliterated by some attack of disease, perhaps inflammation, without, however, remaining engorged, and after having lost the power of dilatation.'"

We regret that he makes no mention here of Dr. Gerhard's valuable paper on the pneumonia of children, published in 1834, in the American Journal of the Medical Sciences. This, be it remembered, was four years previous to the issue of the treatise of MM. Rilliet and Barthez. In that paper, second series, Dr. Gerhard accurately analyzed the symptoms, physical signs, and anatomical appearances of twelve cases, occurring in children between two and six years of age, of what he then styled pneumonia, but which were, without doubt, cases of bronchitis combined with collapse of the lung. The results he obtained were highly interesting and unexpected. The essential nature and cause of the lesion found in every instance, and to which he gave the name of lobular induration, were evidently no more understood by him than was carnification by MM. Rilliet and Barthez. It is now known that Dr. Gerhard was too exclusive in his restriction to children above six years of age, of pneumonia, as it is found in adults. With this reservation, his paper, by far the best of its time, may be consulted even now with advantage.

Of Pneumonia, Dr. Meigs makes two divisions, lobar and partial, preferring the latter term to that of lobular for obvious reasons. During the last four years he has observed 37 cases. Of these 33 were primary and 4 secondary; 19 occurred in children under five years of age; 18 between five and eleven years. In two cases there was double pneumonia, unilateral in all the others; 13 had the upper lobe affected, and 20 the base; of 50 cases, 37 of which were observed during the last four years, two died. The treatment is the same as that given in the first edition.

The subject of Scarlet Fever seems to be a favorite with Dr. Meigs, as he has devoted sixty-four pages to its consideration. He discards, we think without good reason, the old distinctions of simple, anginose and malignant, and substitutes for them the terms mild and grave, according to the severity of the disease. Of the former, mild, he has had 144 cases; of the grave 41, of whom 23, or 57 p. ct. died. The whole subject of scarlet fever, its complication, sequelæ, and treatment are fully and elaborately discussed.

In closing our brief notice of Dr. Meigs' work, we have great pleasure in bearing testimony to its high merit, and especially to the improvements introduced into the present edition. There is certainly, now, no work in the language in which the leading diseases of children are more agreeably, clearly, and faithfully delineated. And, in expressing our regret that some affections peculiar to childhood, have been unnoticed, we feel bound to admit the force of the author's apology for the subjects untouched—"the want of time and opportunity to make use of the materials he has collected." In a succeeding edition, "he hopes and expects to fill up the omissions of the present one;" and we have little doubt that the fulfilment of this pledge will be called for at an early day.

---

*Practical Observations on Aural Surgery and the Nature and Treatment of Diseases of the Ear. With Illustrations.* By WILLIAM R. WILDE, Fellow of the Royal College of Surgeons in Ireland, Surgeon to St. Mark's Ophthalmic Hospical, etc., etc. Philadelphia: Blanchard & Lea, 1853, pp. (with Index,) 475.

Of this valuable monograph we may most unhesitatingly say, in the language of the American editor, "that it will fill a void in our medical literature which has long been felt, and which no work published in this country has ever been adequate to fill." Aural Surgery has, indeed, as the author well observes, been long encompassed with a shroud of quackery, medical as well as popular; and we hail, with no ordinary satisfaction, the appearance of a work, in which a successful attempt has been made, "to lay down just principles for an accurate diagnosis of Diseases of the Ear; to rescue their treatment from empiricism, and found it upon the well established laws of modern pathology, practical surgery, and reasonable therapeutics."

The qualifications of Mr. Wilde for the proper discharge of the task which he undertook in the Treatise before us, are thus graphically portrayed by his friend and former pupil, the American editor, Dr. Addinell Hewson:

"Possessed of extraordinarily quick perceptive faculties, highly cultivated by early discipline and use, of mature judgment and consummate skill, of untiring zeal and industry, and of high literary attainments, few surgeons have had better opportunities, or could have made better use of them, than Mr. Wilde. St. Mark's Hospital for Diseases of the Eye and Ear, the field of his public labors in these branches, is an institution of his own creating, which has been in operation nearly ten years, and is now one of the largest and best conducted of the kind in Great Britain. All those who have had the good fortune of attending his clinics, and observing his practice there, will willingly bear testimony to their admiration of his talents."

We should be glad to be able to quote at length Mr. Wilde's forcible allusions, in his Introductory chapter, to the reprehensible neglect with which the subject of aural diseases has so long been treated by medical men. Our management of this class of affections has, indeed, been characterized by an empiricism, of which we should feel heartily ashamed in prescribing for the lesions of other organs of the body. We have been content, (as Mr. Wilde remarks, with a justness which we are satisfied our readers will generally acknowledge,) "theoretically to believe, and practically to observe, that we know nothing about the diseases of the organs of hearing." And we fear, that but too many of us must recognize a faithful portraiture in his witty description of the ordinary routine of practice in such cases:

"First syringing with hot water and soap, either Castile, soft, yellow, or old brown Windsor, *in the hope* that the deafness or noise in the ears might arise from a collection of hardened wax;—then setting the digestive organs to rights by purgation, and 'a course of bitters,' lest the affection might be 'owing to the stomach.' . . . . . . "Next in order, blistering behind the ears is tried, in order to draw away some peccant humor, that had, perhaps, accumulated round the delicate organ of hearing. These and such like methods failing to give relief, stimulants, often of a very acrid nature, are poured into the external auditory passages, either to restore the reaction,—under the impression that what is a mere attending symptom is the disease,—or to excite or rouse the dormant nervous power; and hot tinctures, turpentine, creasote, and pungent essential oils, are applied to the external surface of the tympanal membrane without mercy. Some practitioners resort to more palliative means, recommending warm almond oil to be dropped into the ear at bed time, or *eau de Cologne* to be rubbed upon the side of the cheek adjoining the auricle, at the same time advising a little black wool to be retained in the meatus, in order to preserve the organ free from cold. To give, however, fair play to the latter remedy, it should be prescribed in full, and according to the old popular superstition, but one which is still extensively resorted to,—the wool should be procured from the left fore-foot of a six years' old black ram! Some advise a slice of fat bacon to be inserted into the meatus every second night; and glycerine is now the fashionable remedy. All these means having failed to give relief, the patient is frequently recommended—an easy mode of getting rid of him—to give galvanism and electricity a fair trial; and if they do not succeed, change of air and scene, sea-bathing, or a 'course of waters' at some of the fashionable places of resort for that purpose is prescribed. Despairing of relief from the legalized practitioner, and getting disheartening opinions from men of eminence and repute, we need not wonder that suffering patients throw themselves into the hands of quacks and nostrum-mongers."

Mr. Wilde's First Chapter is occupied with an extended and very interesting *Bibliography* of aural surgery. Among modern investigators he particularizes Mr. Toynbee as entitled to the first place.

"The labors and investigations of Mr. Toynbee have effected more for aural pathology than those of all his predecessors either in England or on the Continent. He commenced at the right end, and has travelled in the proper direction. He has brought to bear upon the subject the true principles of science, and with the assistance of the microscope,—the aid of every modern artistic appliance to assist him,—accustomed to habits of minute dissection, patient research, and careful observation,—he has accumulated a mass of facts upon the morbid anatomy of the organs of hearing that must lay the foundation for a more rational mode of treating the diseases of those parts than has heretofore been resorted to. Mr. Toynbee has already recorded the results of the dissection of the ears of about 750 persons sent to him for examination, but of which number not more than sixty or seventy were from persons the history of whose deafness was known.

Mr. Toynbee has labored extensively, and with effect, to discover and describe the post mortem appearances which disease has produced in the organ of hearing; and I trust he will long continue to prosecute, with the same avidity, the same honesty of purpose, and an equal amount of critical acumen, his valuable researches. Morbid anatomy, however, is one thing—pathology another. The dead subject upon the dissecting-table teaches the student not disease, but the results of disease. It avails little that the hospital pupil should have pointed out to him, in the dead-room, the violence which sudden accidents may have caused, or the ravages which slow disease has produced in the various organs or textures of the body; it matters not to what extent the microscope may exhibit the wide-spread lesion, or chemical tests disclose morbid products, unless the cases have been observed during life, and the progress of disease previously noted at the bedside. Therefore it is that the School of Vienna,—where a dozen bodies from different parts of the Great Hospital, but the histories of which are unknown, are sometimes cut up, and their post mortem appearances displayed in the lecture-room on a morning,—may, under the able teaching of Rokitansky, Engel, and others, teach morbid anatomy (pathology so called), but does not produce many practical physicians.

Mr. Toynbee's researches prove the position which I long ago advanced, and which from year to year I have been in the habit, not only of teaching theoretically, but practically demonstrating in my clinical lectures,—that the great majority of diseases of the ear producing deafness have their origin in inflammation of one kind or another, Every day's experience confirms me in this opinion; and the cases which I now publish, will, I think, corroborate that view of the subject. Mr. Toynbee has not yet published any separate work upon the ear, but has contributed his observations on the Anatomy, Pathology, and Treatment of the Organs of Hearing to the different societies, and also to the periodicals of London. Among the latter may be mentioned chiefly, the Philosophical and Medico-Chirurgical Transactions, from 1843 until the present date. He has also written several valuable papers in the Medical Times, Lancet, Provincial Journal, and Edinburgh Medical Journal."

A considerable space in the Bibliography chapter is devoted to the "*Glycerine Cure*," which Mr. Wilde considers as very much of a humbug. And immediately following his exposure of the worthlessness of this fashionable remedy, we find some apparently well-merited strictures on a charlatanism which has had some run in England, viz., Dr. Turnbull's cure of deafness by the application of veratria to the external meatus and the parts joining the auricle. Mr. Wilde's manly tone on the subject of the aural quackery of the day, within the ranks of the profession, is highly commendable.

Chapter II. relates to *The Means of Diagnosis.* In exploring or operating upon the external auditory passages, we find that he employs the *tubular* specula of Gruber, of Vienna, which are merely "conical silver tubes of different calibres, an inch and a half long, five-eighths of an inch wide at the greater aperture, and varying from two to four lines in the clear at the smaller extremity." In using the specula, he prefers direct sun-light to all artificial illumination, and discards all the instruments with prisms, lamps, &c., as unnecessary. Attempts to dilate the auditory passage by means of instruments, Mr. Wilde justly characterizes as "showing a want of anatomical knowledge in their inventors, for the most any speculum can effect is to straighten the external cartilaginous portion of the tube, and thereby allow the light to play upon the interior."

In connection with the subject of *Eustachian catheterism*, Mr. Wilde (after describing the instrument which he is in the habit of using, and the mode of applying it,) holds the following cautionary language:

"I know few subjects upon which there is more general ignorance than the value to be attached to Eustachian catheterism, or the best mode of employing it. Some writers would lead us to suppose that this operation is of use in a far greater number of aural diseases than, according to my experience, is the fact. In order to facilitate our diagnosis, they would have us explore the middle ear by the air *douche*, a jet of water, or a solid instrument, in almost every case that presents. Errors of commission are, in both medicine and surgery, I believe, of much greater injury than those of omission; and the introduction of a foreign body into the Eustachian tube, forcing a volume of cold air, or injecting a quantity of fluid, even warm water, into the cavity of the middle ear, as some recommend and say they practise, is in nearly all cases, while inflammatory action is going forward in the parts submitted to the process, unnecessary and frequently injurious. Again, the mode in which I have heard both patients and bystanders speak of instruments—catheters of various kinds, gum-elastic, and metallic, and even solid sounds, some of the latter much larger than the bore of the aural end of the Eustachian tube even in the dry bone—being introduced into the middle ear, in order to explore that cavity, to wash out its contents, or to break up collections of mucus within it, or to dilate strictures and contractions of the tube itself, convinces me that the desired object was not, fortunately for the sufferers, obtained. Even Kramer speaks of pushing a catgut bougie, introduced through the Eustachian tube, "between the handle of the malleus and the incus !" People are, however, beginning to find that this attempt—for I know in many cases it is only an attempt—to force solid or fluid substances into the cavity of the drum, is as ineffectual to remove deafness as the almost indiscriminate excision of the tonsils—since preserved in pickle-pots—recommended for a like purpose a few years ago, has proved to be. I have heard of cases in which the middle ear has been said to be explored by such mechanical means, even in this country, and I have been shown steel sounds manufactured for the purpose. Such instruments are, however, with the exception of the tearing and inflammation which they may cause in the nasal extremity of the tube, harmless, for they could not by any possibility, even in the dead subject, be passed through the upper end of the Eustachian tube. We should bear this rule ever in remembrance before exploring the middle ear; it is one which Sir A. Cooper observed to me many years ago, and I have ever since acted upon it:—Whenever the patient is himself able to inflate the tympanum, never use any artificial means to do so; it is unnecessary and may be injurious. Let me to this aphorism add another, to which I have already alluded, and which surgeons would do well to remember. Where there is reason to believe that the cavity of the drum is inflamed, carefully abstain from all poking with catheters, or any attempt to introduce foreign substances into that delicately-organized portion of the animal machine. As good general surgery teaches us to avoid the introduction of instruments through an inflamed urethra, or into an irritable bladder, so ought judicious aural surgery to teach us to abstain from meddling in the cases to which I have alluded. The only solid instrument with which I now ever venture to explore the Eustachian passage, and that only for a short distance, is an ivory bougie, rendered flexible by having the earthy matter removed by immersion in acid, and the point of which for an inch at least had been previously softened in water so as to resemble a piece of gelatine. A large size catheter should be first introduced, and the bougie passed up through it; but stricture of the Eustachian tube is so exceeding rare, and so difficult to recognize during life, that the surgeon is very seldom called on to practise such an operation."

Chapter III. is devoted to Statistics and Nosology of Ear Diseases. The author here takes the opportunity, in alluding to Kramer's statistical chart and tables on this subject, to express the most unqualified disbelief in the "nervous deafness," to which the Berlin aurist attributes more than half his cases. He contrasts the results of his own observations, "faithfully taken as they presented at a public hospital, in the presence of, and equally observed by, a number of medical men and intelligent students," with the figures afforded by Dr. Kramer. And, while not denying the great prevalence of nervous deafness, he doubts decidedly the proportion which, according to the German aurist's belief, they bear to the entire mass of diseases of the organ of hearing.

The remainder of the book is of a practical character, and the subjects discussed are in the following order:—1. Diseases of the Auricle, Mastoid Region, and External Meatus. 2. Diseases of the Membrana Tympani. 3. Diseases of the Middle Ear and Eustachian Tube. 4. Diseases of the External Ear. 5. Otorrhœa. We may particularize here as points treated with especial interest, the management of strumous myringitis, (p. 263)—that of accidental perforation of the membrana tympani, (p. 287)—the discussion of the operation of extirpation of the tonsils, (which Mr. Wilde condemns,) (p. 351)—and of the subject of nervous deafness, (p. 361).

In the Appendix, the subject of Deaf-Dumbness is noticed at considerable length, and will well repay perusal. The author does full justice to the efforts of our country in this direction. We quote his remarks with great pleasure:

"We now turn to America, where the claims of the deaf and dumb have not only met with a hearty response from the public, but where the country at large has made ample provision for their maintenance and instruction. So early as 1793 there appeared in the Transactions of the American Philosophical Society, 'Cadmus, or, a Treatise on the Elements of Written Language, and with an Essay on the Mode of Teaching the Deaf or Surd, and, consequently, Dumb to speak.' As I have already mentioned, the number of instances in which national institutions have had their origin in the interest awakened in some philanthropic individual by a mute child, is remarkable. In 1807, the deaf and dumb daughter of Dr. Cogswell, of Hartford, U. S., attracted the attention and enlisted the sympathies of the late Rev. Thomas H. Gallaudet,—a man who united the true principles of charity with distinguished scholarship and unwearied assiduity and patience. He subsequently proceeded to Europe, in order to acquire the art of imparting instruction to the deaf and dumb; and having remained for some time a pupil under the Abbé Sicard, he returned to America, accompanied by M. Clerc, who was then (in connexion with Massieu, the favorite pupil of De l'Epeé) one of the mute assistants of the Parisian institution.

The first institution for the instruction of deaf mutes in America was opened at Hartford in 1817. Since then, no less than twelve other large establishments for a similar purpose have been erected in that country; and the expenses in these are defrayed, like most of those on the Continent of Europe, by a state provision, either in grants of land or money.

The subject of muteism generally, as well as the system of instruction in America, has engaged the attention of the learned and benevolent, and the publications relating to muteism which issue from the press in that country are numerous and valuable. The reports which emanate from the American Institutions, particularly that at New York, are models of their kind. The Rev. W. Gallaudet published some sermons on muteism, a 'Child's Picture, Defining and Reading Book' (Hartford, 1830,) and also some reviews and literary articles relating to the deaf and dumb. Several interesting addresses on that subject have been also delivered on special occasions by Laurent Clerc, Dr. Mitchell, Mr. Lewis Weld, and Harvey Peet. These were subsequently published, and contain most valuable information. Mr. Peet has also written several school-books and elementary works for the deaf and dumb; and to the courtesy of that gentleman I am largely indebted for much information regarding the American institutions, and for an extensive collection of American works relating to muteism. The North American Review for 1834 has a philosophical and admirably written article upon the education of the deaf and dumb, and the Philadelphia edition of the Encyclopædia Americana likewise contains an article on the dumb and deaf. The Christian Spectator for 1837, the New York Literary and Theological Review for 1835, and the Biblical Repository for 1842, also contain articles on the existing state of the art of instructing the deaf and dumb. Tales and poems bearing upon the subject of muteism have likewise appeared from the pens of Mr. Nack, Mr. Burnett, and Mr. John Edwin Mann,—the latter a deaf mute, educated at the Hartford Asylum.

In October, 1847, appeared the first number of the American Annals of the Deaf and Dumb, originally conducted by the instructors of the American Asylum, and afterwards ably edited by Mr. Luzerne Rae,—a quarterly periodical which continues to the present time. This is by far the most valuable publication connected with the subject on which it treats at present in existence."

The additions of the American editor, (who modestly declines the usual editorial distinction of a place in the title-page,) are valuable and judicious. He has appended to the author's observations on Syringing, an account of a useful instrument invented by Dr. Hullihen, of Wheeling, Va., to enable patients, without aid, to syringe out the external meatus for themselves. On the subject of counter irritation, he recommends and describes the *Cantharidal Collodion*, as a desirable substitute for the ordinary blistering ointment. Malformations of the ear are illustrated by the editor, with an interesting case of Professor Mussey's, of "congenital absence of the meatus auditorius externus of both ears, without much impairing the hearing." Other interesting cases, extracted from American journals, are appropriately introduced by Dr. Hewson; and we notice also a very good manipulative suggestion, from the same source, for the application of caustic solution to the mouth of the Eustachian tube.

The wood-cut illustrations of this work are exceedingly well done; and its general appearance is of the highest style of American publications. It is, emphatically, a good book.

---

*A Treatise on Operative Ophthalmic Surgery.* By H. HAYNES WALTON, F. R. C. S., Surgeon to the Central London Ophthalmic Hospital, &c., &c. *First American from the First London Edition. Illustrated by One Hundred and Sixty-nine Engravings on Wood.* Edited by SQUIRE LITTELL, M. D., Author of a Manual of the Diseases of the Eye, Surgeon to Wills Hospital for the Eye and Limbs, &c. &c.—Philadelphia, Lindsay & Blakiston, 1853. 8vo. pp. 599, (including Index.)

The remarkably handsome style in which the American edition of this attractive book has been produced, does great credit to its enterprising publishers. Some acquaintance with the London original had disposed us to look with interest for the present reprint, and a closer examination of the latter has more than fulfilled our previous expectations. The notes and corrections of its distinguished editor, taken in connection with its near approach to the English copy in engraving and typography, have rendered it, what reprints always ought to be, decidedly more desirable than its foreign prototype. At all events, the manner in which Dr. Littell has been seconded by the publishers in the getting up of the volume, materially enhances the value of his share of the undertaking, and must rapidly secure the favorable reception which he anticipates in his preface, and considers so abundantly justified by the intrinsic merits of the treatise.

These merits are so clearly and faithfully set forth in a few words of this preface, that we cannot do better for our readers, or the book itself, than to insert them here.

"It is evidently the production of a thoughtful and independent observer, thoroughly acquainted with his subject, and capable of imparting to others, clearly and plainly, the knowledge which he possesses. The information it contains is, for the most part, accurate and precise, and its preceptive teachings practical and judicious. The reader is not perplexed by the necessity of determining for himself the relative value of different modes of procedure; but is authoritatively instructed in that which experience has proved to be the most appropriate for the accomplishment of his purpose. The sound principles of pathology which it inculcates, derived from the best schools of English Ophthalmology, naturally lead to a rational and conservative treatment. It embodies the results of extensive literary research and close personal observation, under circumstances of rare opportunity; and though of unequal excellence—some parts being more carefully written and more fully developed than others, with occasional discussions also of topics not strictly included in its title—is alike creditable to the author, and a valuable contribution to ophthalmic surgery. It has been very favorably noticed by the leading medical journals of Great Britain, and is the subject of especial commendation by a distinguished authority. 'In the whole range of ophthalmological literature,' says Dr. Mackenzie, in a review which is not sparing of criticism, 'we know of no work that, on the whole, better deserves a place in the library of the surgeon. It is full of sound and practical views, and shows the rapid advances which are being made in this department of the medical art. Most of the cases have occurred to the author himself, and prove him to be an observing and able practitioner. His style is good, being perspicuous and unaffected; and any errors in the book are few and trivial in comparison of the mass of accurate information which it contains.'"

Mr. Walton does not confine himself to a logically strict discussion of the mere operations on the eye and its appendages. He naturally avails himself of the elastic meaning of his title, and treats his readers to something more agreeable and useful than a dull routine account of mere manipulations, which of themselves comprise but a subordinate part of the whole range of operative ophthalmic surgery. In fact, like most of the standard issues from the British press, our author's treatise appears to be much more practical and substantial, than it is formally precise and systematic. While he does not affect to exhaust his subject, either in details or outline, he has nevertheless supplied the working practitioner with an amount of available instruction, in regard to the surgical treatment of the eye, that will not easily be displaced by the elaborate teachings of much more voluminous guides. We are not inclined, therefore, to complain because in treating of operations on the eye he has incidentally favored his readers with a great deal of interesting information on the pathology, diagnosis and therapeutic management of several injuries and diseases of the visual organs. We consider this liberal treatment of his subject one of the best points of his book, and one that cannot fail to prove in it a great source of popularity, as well as actual utility among the profession of this country.

The twenty-one chapters treat successively—1st. Of the use of chloroform in Ophthalmic Surgery; 2. Of Ophthalmic instruments in general; 3. Of injuries; 4. Of foreign bodies on or in the eye, larvæ under the lids, ossification of the occular tissues; 5. Affection of the eye lids, and of the lachyrmal passages; 6. Affections of the lachrymal outlets; 7. Caries of the orbit; 8. Nævi and vascular tumors; 9. Scarification in chemosis; 10. Strabismus; 11. Tumors; 12. Protrusion of eye-ball; 13. Staphyloma; 14. Conical cornea; 15. Removal of opacities; 16. Cataract; 17. Entozoa; 18. Artificial eyes; 19. Malignant affections; 20. Artificial pupil; 21. Extirpation.

The chapter on the use of chloroform, presents a new feature in the study of Ophthalmic Surgery. The account of it in this instance is clear, concise, and sufficiently full for practical purposes, although we confess that we should have been glad to find some kind of exposé of the positive or approximative therapeutic results from operations under chloroform and other anæsthetic agents. Anæsthesia is still regarded here as a complication not to be desired in most operations on the eye; and we want something more assuring than even Mr. Walton's great experience, to satisfy our doubts as to the ultimate advantages of such an almost indiscriminate resort to chloroform, as he would seem to advocate. "Except the operation for the extraction of cataract," he says, "or the division of the cornea, to a like or nearly equal extent, for any other purpose, there is no operation on the eye in which well-founded objections exist to the previous use of chloroform, or ether, as anæsthetic agents." The objection arises, in the cases mentioned as exceptional, from the risk of vomiting and consequent loss of the vitreous humor. This danger of vomiting affords, in our view, a serious objection to the use of anæsthetics in many other operations not included in the above exceptions. At any rate, the question to be answered is *cui bono* in many of the comparatively slight and easily tolerated surgical procedures which have to be performed. We can readily appreciate its value in operating upon children or timid patients, or upon very sensitive or irritable adult eyes; but beyond this, we think, more definite rules of action are needed for our guidance in the resort to it, than Mr. Walton has thought proper to lay down.

Chapter 2d is occupied with the consideration of ophthalmic instruments in general, upon which it presents us with some excellent remarks. Lightness, small size, and good order as to points and edges, are dwelt upon as indispensable requisites in all these implements. Cataract needles, knives, and the like, should have wooden handles, small, round and smooth, in preference to the old fashioned and many sided ivory holders generally employed. The wood-cuts in this chapter are, like the illustrations throughout the book, appropriate and admirably done. Here and elsewhere, they facilitate most effectually the precepts and descriptions of the text, and constitute one of the strongest attractions of the volume.

The third chapter is one that might not be looked for in a dissertation on operations, since it is devoted to injuries from mechanical or chemical agents. It contains a good deal of instructive matter, and many useful hints; so that whether out of place or not, it must prove acceptable to the great majority of readers, and could not conveniently be spared. Chapter fourth, like its immediate predecessor, is not wholly occupied with operative surgery, and is none the less valuable and interesting, being principally engaged with the subject of foreign bodies in the cornea and within the ball. The number of cases detailed in these two chapters, renders them in the highest degree useful to practitioners of every grade, all of whom are liable to the emergencies which are here forcibly described and illustrated.

The next chapter, which is a capital one in all respects, treats at length of various affections of the eye-lids, such as abscess, symblepharon, anchyloblepharon, epicanthus, ptosis, trichiasis, entropium, entropium with trichiasis, ectropium, obstruction of the meibomian ducts and conjunctival calculi. This is followed by an interesting chapter on affections of the puncta, canaliculi, and what he calls the lachrymal tube; by a chapter on caries of the orbit; by another, very carefully prepared, on nævi materni and venous and arterial aneurismal growths; by one on incision of the chemosed conjunctiva in purulent ophthalmia, and by another on strabismus. Then we have a well furnished chapter on tumors of the eye; another, also good, on protrusion with anæmia and enlarged thyroid, and on exophthalmia from other causes, on rheumatic inflammation of the occular sheath, periostitis, abscess and inflammation within the orbit, and disease of the optic nerve. Next follow three shorter chapters, devoted respectively to staphyloma and its attendant characteristics and remedial management, to conical cornea, and to the treatment by operation or otherwise, of opacities of the cornea. In discussing the various topics above hastily enumerated, Mr. Walton, without presenting much that was actually new, has managed to enrich his pages with many highly interesting cases, and a great deal of pertinent matter, which, accompanied and qualified as it occasionally is by the comments of his American editor, will afford the practical inquirer more material aid than could be gleaned in the same time, from any other work yet extant on the subject.

Our limits forbid the notice of many points, which in a more elaborate review would suggest themselves to our attention. We might dwell with advantage upon several questions of theory and practice, as well as upon some important observations—such, for example, as those concerning trichiasis and entropium, their causes and best mode of cure, the phenomena of exophthalmia, the effects of certain conditions of the lens in staphyloma, the treatment of nævi and vascular tumors, the history and management of other morbid growths, the ablation of opacities, &c., &c.—but we are obliged to hasten to a close, with an equally brief glance at the remaining six divisions of our author's task.

The first of these, (chap. xvi,) is a long and able one on cataract. The whole subject is here considered *in extenso*, and with a praiseworthy attention to matters of practical detail in the previous and subsequent care of the patients, as well as in the different stages and modes of operating. We are glad to find the needle recommended by Mr. Walton, and represented in the marginal engraving, to be substantially the same as that preferred by the operators at the Wills Hospital in this city, and which has been employed with so much success (about 80 per cent cured according to Dr. Littell) upon the patients of that house.

Chapters xvii and xviii are occupied with entozoa and cysts within the eye and its appendages, and with artificial eyes. Chapters xix and xx, the one on malignant diseases of the eye, and the other on artificial pupils, are two long and admirable papers, which are decidedly the best of the book. Chapter xix especially, in its whole character, arrangement, matter and sound conservatism of doctrine and precept, commends itself to the attention and respect of every reader, and can not fail to secure an honorable position to the whole work, independently of all other claims, Chapter xx, on artificial pupil, is scarcely less worthy of approval for clearness and beauty of arrangement and illustration, and for the interest and variety of the cases cited in connection with the different questions belonging to this important subject. The conditions under which an operation may be undertaken, and those which contra-indicate it, the relative advantages of the several positions in the iris for a pupil, the size and shape of the pupil, the classification of the principal morbid states of the eye requiring a pupil, with the most appropriate operations—are all handled in the very best manner.

The only remaining chapter treats of extirpation of the eyeball, and of the entire contents of the orbit. We take our leave of our author and his editor, with a quotation which neither will regret to see again in print, and which we place before the readers of this journal, rather for the sake of the solemn lesson it conveys, than as a specimen of Mr. Walton's style :—

"All that we have hitherto ascertained as to the origin and nature of cancer, leads us to the conclusion that it is a constitutional, not a local disease, and that the tumor or sore is merely the evidence of the poison that is at work within; the outlet, so to speak, at which the *materies morbi* endeavors to escape from the system. If this be true, it is evident that any attempt to arrest the disease by the removal of its local manifestation can be attended only with disappointment; and that the illustration adduced by Mr. Simon that we might as well attempt the cure of gout by the amputation of the offending toe, is, in many respects, strictly applicable. I do not mean to assert that cancer, when left to run its course, is inevitably fatal; or that an operation for its removal is invariably followed by its recurrence; cases occur, from time to time, in which it disappears spontaneously from the system, by processes which it is not necessary here to describe; and several well-authenticated instances are on record, in which the extirpation of malignant growths, even under the most discouraging circumstances, have been followed by complete and permanent recovery. Such exceptional cases, however, are so rare, and the conditions under which they occur are so little understood, that it would not be safe to make them the basis of any practical conclusion." (p. 536.)

It is gratifying to meet with views of this high stamp, in regard to questionable operations, in a work which is destined, under its peculiar auspices, to exert a considerable influence on the ophthalmic practice of the country. We take the greater pleasure, therefore, in recommending it as a safe and convenient adviser which ought to have its place in every medical library,—not as a substitute for Hays' Lawrence, and Littell's manual, but as an admirable and most efficient companion to those indispensable monitors.

*The Practice of Surgery.* By JAMES MILLER, F. R. S. E., F. R. C. S. E., Surgeon in Ordinary to the Queen for Scotland; Surgeon in Ordinary to his Royal Highness, Prince Albert, for Scotland; Professor of Surgery in the University of Edinburgh; Consulting Surgeon to the Royal Infirmary; &c., &c., &c. *Third American, from the Second Edinburgh Edition. Edited, with additions,* by F. W. SARGENT, M. D., one of the Surgeons to Wills Hospital. *Illustrated by Three Hundred and Nineteen Engravings on Wood.* Philadelphia, Blanchard & Lea, 1853. pp. 720.

Notwithstanding the numerous treatises of a practical character which have lately been issued from the press, this edition of the Practice of Surgery of Prof. Miller, cannot fail to receive an extended circulation. It is the third American from the second Edinburgh edition, a fact which must be highly gratifying, not only to the publishers in this country, but to the author also, as an evidence of the appreciation of his labors on this side of the Atlantic.

Many subjects are treated of in this edition, which were not included in the last, and its numerous illustrations will, we have no doubt, render it a far more acceptable volume. Indeed, the student of surgery will find in the "Principles" and "Practice" of Surgery of Professor Miller, (to use the author's modest phraseology,) "a tolerably complete system of surgery."

The American editor, Dr. Sargent, has appended many valuable notes and introduced numerous additional illustrations, which greatly enhance both the appearance and usefulness of the work.

We are glad to see, that the Bibliography, which was omitted in the former editions, is now restored; and we feel also called upon to speak in high terms of the mechanical execution of the book. It must maintain a prominent position as a standard text-book in our medical schools.

---

*The Maternal Management of Children in Health and Disease.* By THOMAS BULL, M. D., member of the Royal College of Physicians, author of "Hints to Mothers" for the Management of their health during Pregnancy and the lying-in room. *Second Edition.* Philadelphia, Lindsay & Blakiston, 1853.

A strong dislike to works on popular medicine may be said to have existed in the profession from the early period of Buchan's "New Family Physician," to the present time. A meddlesome and opinionated state of mind, which shows itself in words and deeds, is frequently created in those who use such works, and this is not only annoying and disheartening to the practitioner, but has often led to dangerous and even fatal consequences.

Such are the arguments of those who object to popular treatises on medicine. On the other hand, humanity, the eradication of old prejudices and superstitions, the inability of obtaining medical advice, and the protection of the ignorant public against the impositions of quackery, are brought forward by the writers as pleas in satisfaction or in extenuation of their offence. We shall sum up judicially by saying, after worthy old Sir Roger, "that a great deal may be said on both sides of the question."

Dr. Bull makes three divisions of his subject:—"The management of children in health, the management of children in disease, and the prevention of scrofula and consumption." For many reasons, we prefer the first and last sections.

The style is plain and unpretending, and the advice sound and sufficiently in detail. Where such books are needed, it may be safely recommended.

*Dr. Hooper's Physician's Vade Mecum: or a Manual of the Principles and Practice of Physic. Considerably Enlarged and Improved.* By WILLIAM AUGUSTUS GUY, M. D., &c. *With Additions.* By JAMES STEWART, A. M., M. D., &c. Philadelphia. E. Barrington & G. D. Haswell, 1853. pp. 541. (*With Index.*)

This is one of the oldest and most popular of the "Manuals." It was thoroughly remodelled some years since, under the able editorship of Dr. Guy, and may now be safely commended to the student as a very complete, clear, and accurate résumé of the "Principles and Practice of Physic."

---

*A Manual of Obstetrics.* By THOMAS F. COCK, M. D., Physician to the New York Lying-in Asylum, to Bellevue Hospital, etc. New York. Samuel S. & William Wood, 1853. pp. 250. (*With Index.*)

A hasty glance at this handbook has impressed us very favorably of its merit. The author "makes no pretensions to originality," in its behalf, but puts it forth as a compilation, the basis of which has been his notes of the lectures of Professor Gilman, of New York.

---

*Hallucinations: or the Rational History of Apparitions and Visions, Dreams, Ecstacy, Magnetism and Somnambulism.* By A. BRIERRE DE BOISMONT, Docteur en Médecine de la Faculté de Paris, Directeur d'un Etablissment d'Aliénés, etc. etc. *First American, from the Second Enlarged and Improved Paris Edition.* Philadelphia, Lindsay and Blakiston, 1853. pp. 553.

This book contains an immense mass of curious and interesting cases on the subjects indicated by the title, which must give it great value as a work of reference to the student of these topics.

The curious in such matters will find in it a most careful résumé of all the "authentic (?) facts" connected with the subject in question, classified and subdivided on scientific principles, and studied in relation to their "symptoms, causes, lesions, prognosis, diagnosis and treatment."

In the discussion of these topics, the author puts forth much acute and occasionally deep speculation; but there is a vein of combined credulity and mysticism throughout the book, incompatible with claims to a place among philosophical dissertations on the subject of mental pathology. M. De Boismont distinctly avows his "sympathy with the grand creed of supernaturalism," and is, moreover, a believer in clairvoyance. He considers the fact established, "that persons and objects, in connection with the person magnetized, can be seen by him under their real forms;" and believes "the phenomena of clairvoyance, prevision, and second sight to depend on a sudden illumination of the cerebral organ, which calls into activity sensations that have hitherto lain dormant."

The translation (from an anonymous pen,) is spirited and agreeable. Occasional inaccuracies, (such as we *will*, constantly, for we *shall*; the *Port* for *Oporto*, p. 65; &c.,) are noticeable; and we feel bound also to hold the translator responsible for allowing such blunders in English history to stand uncorrected, as "the Marquis of Londonderry, *afterwards* Lord Castlereagh," and "the *Harrowby* ministry."

---

*The Prescriber's Pharmacopœia:* Containing all the medicines in the London Pharmacopoeia. *Altered to correspond with the U. S. Dispensatory. Third American from the Fourth London Edition.* By Thomas F. Cock, M. D. New York, S. S. & W. Wood. pp. 178, (with Index.)

This will be found a most useful little pocket companion to both the student and practitioner, containing in a very small space a great amount of varied information, on subjects of every-day importance.

---

*On the Use of Chloroform in Midwifery.* By George N. Burwell, M. D., one of the Physicians of the Buffalo Hospital.

*Fracture Tables.* By Frank H. Hamilton, M. D., Professor of Surgery in the University of Buffalo.

For these highly valuable statistical pamphlets, we are indebted to two distinguished physicians of the accomplished profession of Buffalo; a city which is rapidly becoming elevated by the energy and talent of her leading physicians, to the position of a medical centre.

Dr. Burwell gives the statistics of one hundred and eighty cases, in which he administered chloroform for the relief of labor-pains. He does not discuss the *propriety* of administering this agent; but, waiving argument on that point, he gives us, first, his statistical results upon the most important points of the cases in which he administered it; secondly, an account of its effects, as noticed by him, upon the symptoms and progress of labor; and, thirdly, his mode of exhibiting it, the doses in which it may be safely given, and the rules by which he is governed in its use.

Dr. Hamilton's *Fracture Tables* are an important addition to the statistics on this subject.

---

*Transactions of the Tennessee State Medical Society, at their Twenty-fourth Annual Session, convened at Nashville, May* 4, 1853.

This volume contains, with the proceedings of the Tennessee State Medical Society, an interesting address on the history of Medicine, by Dr. J. M. Watson, President; a valuable report on the adulteration of medicines, by Dr. R. O. Currey; reports of Committees, by Drs. W. P. Jones and G. B. Haskins; and cases by Drs. Ramson, Park, Knight, and Bowlin. The Transactions are most creditable to the Profession of Tennessee.

---

*The Physician's Visiting List, Diary, and Book of Engagements, for* 1854. Philadelphia, Lindsay & Blakiston.

This most convenient little work has already been issued, for 1854, by the enterprising publishers. Several improvements have been made in it, and it has been simplified by the withdrawal of all extraneous matter, except a table of poisons and antidotes. No physician, who has ever had the advantage of so useful a pocket companion, can, we are satisfied, afford to be without it.

*The Microscopist; or A Complete Manual* on *the Use of the Microscope. With Illustrations.* By JOSEPH H. WYTHES, M. D. Philadelphia, Lindsay & Blakiston, 1853. pp. 212 (with Index.)

The value of this useful little manual has been well shown in the rapid exhaustion of the first edition in little more than a year. Numerous important additions have been made to the present edition, which is highly creditable to both the author and publishers.

